# Cardiovascular Organ Damage in Clinical Subtypes of Systemic Sclerosis: Arterial Stiffness and Echocardiography Might Not Be the Ideal Tools for Patient Risk Stratification

**DOI:** 10.1155/2021/7915890

**Published:** 2021-04-23

**Authors:** Eleonora Avenatti, Giulia Bruno, Marta Priora, Simone Parisi, Chiara Ballini, Franco Veglio, Alberto Milan, Enrico Fusaro

**Affiliations:** ^1^Internal Medicine and Hypertension Division, Department of Medical Sciences, AOU Citta' Salute e Scienza of Turin, University of Turin, Turin, Italy; ^2^Rheumatology Unit, AOU Citta' Salute e Scienza of Turin, University of Turin, Turin, Italy

## Abstract

**Background:**

Vascular damage is recognized as a diagnostic landmark in systemic sclerosis (SSc), both in its limited and diffuse subtypes. Early detection at a subclinical stage with transthoracic echocardiography (TTE) and carotid femoral pulse wave velocity (cfPWV) may be helpful in therapeutic planning and management. *Aim of the Study*. The aim of the study was to evaluate presence of subclinical cardiovascular damage in patients with limited and diffuse SSc in comparison with a cohort of healthy individuals.

**Methods:**

Consecutive patients with limited and diffuse SSc underwent complete TTE and cfPWV and a complete review of clinical data. As controls, 23 healthy subjects with similar hemodynamic profiles were selected.

**Results:**

41 patients (35 female, aged 56.9 years), 21 with diffuse and 20 with limited SSc, were recruited. Past medical history, cardiovascular risk factors, gender distribution, and disease duration were similar in the two groups as well as TTE parameters and hemodynamic indexes—cfPWV (6.5 [6–6.8] vs. 7.0 [6.2–8.5], *p*=0.24) and augmentation index (145.6 ± 14.2 vs. 149 ± 20.6, *p*=0.52). Patients with limited SSc were 10 years older than patients with diffuse SSc. In the multiple regression analysis, only age (*p*=0.0154) and disease duration (*p*=0.0467) resulted as the significant determinant of cfPWV. When compared to healthy controls, no significant difference emerged in TTE or hemodynamic indexes.

**Conclusion:**

In SSc, cfPWV increases with age, with no additional impact of pathology or subtype. Vascular damage in the SSc population is not accurately reflected in increased arterial stiffness, as evaluated with cfPWV, or classically defined echocardiographic findings of organ damage (i.e., left ventricular concentric remodelling and increased filling pressures).

## 1. Background

Systemic sclerosis (SSc) is a chronic and progressive connective tissue disease arising as a consequence of altered immunologic processes, with vascular injury, inflammation, and fibrosis of the skin and different internal organs [1]. A “limited” and a “diffuse” subtype of the disease are recognized and differentiated on the basis of different autoantibodies pattern and clinically distinct visceral and cutaneous involvement, with the diffuse form showing a more severe phenotype [2]. Microvascular involvement is one of the hallmarks of the disease, but the damage can occur at every level of the vascular tree [3].

Carotid-femoral pulse wave velocity (cfPWV), the gold standard for noninvasive evaluation of arterial stiffness [4], has demonstrated a specific role in cardiovascular risk stratification independently of other well-known risk factors [5, 6].

cfPWV has been shown to be higher in SSc patients than in healthy, age-matched controls in some [7–9] but not all [9, 10] previous works. The specific role of SSc in promoting arterial stiffening is therefore still unclear, as well as impact of SSc subtypes on macrovascular damage as assessed specifically by cfPWV.

The aim of the study was to evaluate the presence of subclinical cardiac organ damage and arterial stiffness, as a landmark of macrovascular damage, with the current gold standard represented by transthoracic echocardiography and cfPWV in patients with SSc, focusing in particular on differences between limited and diffuse SSc.

## 2. Methods

### 2.1. Patients

Consecutive patients referred to the Rheumatology Department of “Città della Salute e della Scienza,” University Hospital in Turin (Italy), were recruited. All patients met the ACR–Euler criteria for SSc diagnosis and classification [11]. Past medical history, risk factors, and family history were assessed through a detailed anamnestic interview. SSc subtype and clinical and serological data were collected for each patient from their medical records. SSc-specific data—pulmonary function test, EGDS capillaroscopy results, autoantibodies pattern, and renal function—were retrieved from the EUSTAR (European Scleroderma Trials and Research group) database for every patient.

Patients with either limited or diffuse systemic sclerosis were compared to a well-matched control population consisting of healthy, normotensive volunteer subjects (no history of arterial hypertension or antihypertensive treatment, confirmed normal office blood pressure and ABPM values, and absence of hypertension mediated organ damage), free of cardiovascular diseases (such as coronary artery disease, valvular heart disease, aortic disease, atrial fibrillation, stroke, chronic kidney failure, and diabetes), evaluated at the Hypertension Unit of the same institution. The study was approved by the local ethical committee, and all patients gave their informed consent to participate.

### 2.2. Cardiovascular Organ Damage Evaluation

#### 2.2.1. Echocardiography

All subjects involved in the study underwent a complete evaluation of cardiovascular target organ damage with a complete transthoracic echocardiogram (TTE) and with central pressure appraisal and cfPWV measurement.

A two-dimensional (2D) TTE was performed in all patients at rest in the left lateral decubitus position, with a commercially available ultrasound system equipped with a S5 probe for 2-dimensional and tissue Doppler imaging acquisition (iE33, Philips Medical System, Andover, Massachusetts). In brief, left ventricular (LV) mass was calculated from the end-diastolic LV internal diameter (LVIDd), interventricular septum (IVS), and inferolateral wall thickness (ILW) and normalized to body surface area (BSA). Relative wall thickness (RWT) was calculated as (2^∗^ILW)/LVIDd. Patterns of left ventricular geometry were defined according to the ESH/ESC recommendations. LV hypertrophy was defined as LV mass indexed to BSA >95 g/m^2^ in women or >115 g/m^2^ in men. Body surface area (BSA) was calculated using the DuBois and DuBois formula. LV volumes were assessed through Simpson's biplane technique from apical 4- and 2-chamber views and indexed to body surface area and then used to evaluate LV systolic function (LV ejection fraction, EF). Offline analysis and measurements were performed in agreement with the last International Guidelines [12], by expert European Association of Echocardiography-accredited staff.

#### 2.2.2. cfPWV and Ai

In the same day, central pressure appraisal and cfPWV evaluation were performed following standardized protocols. In brief, blood pressure and heart rate were measured three times, at 2 min intervals using a validated automatic oscillometric device (Omron Matsusaka Co., Ltd., Mie, Japan). The mean value from these three measurements was used for further analysis.

cfPWV was measured along the descending thoracoabdominal aorta by the foot-to-foot velocity method, with a dedicated instrument (Sphygmocor system, AtCor Medical, Sydney, Australia) as previously published and validated [13]. With this system, pressure waveforms are obtained with a dedicated transcutaneous transducer applied sequentially over the common carotid artery and the femoral artery. The time delay, or pulse transit time (*t*, in seconds), is measured by subtracting the time between ECG-R wave and proximal (carotid) waveform foot from the time between ECG-R wave and distal (femoral) waveform foot. In consideration of the strong heart rate dependence, PWV values which were obtained with heart rate difference >10% between carotid and femoral sites were discarded. The distance covered by the pulse wave (D, in meters) is supposed to be the surface distance between the carotid and femoral recording sites. Therefore, it is calculated by multiplying the surface distance measured between the two points by a correction factor of 0.8, as previously described in detail and validated [4, 14].

cfPWV is obtained as D/*t*. A cfPWV value > 10 m/sec was considered pathologic [15].

The assessment of the augmentation index and central pressures was allowed by the estimation of aortic pressure waveforms, which were obtained through the measurement of radial artery waveforms, by using a high-fidelity micromanometer (SPC-301; Millar Instruments, Houston, Texas, USA). Radial artery tonometry was performed, and a validated transfer function (Sphygmocor, AtCor, Sydney, Australia) was applied in order to calculate aortic pressure waveforms. The calibration of central artery pressure was obtained by using noninvasively recorded brachial blood pressure as surrogate of radial arterial pressure. This method is based on the fact that mean blood pressure remains constant and diastolic blood pressure does not significantly change along the artery tree [4]. Augmentation pressure was defined as the height of the late systolic peak above the inflection. The aortic or central augmentation index (Ai) was calculated as the ratio of the pressure difference between the “shoulder” of the pressure wave and “peak” systolic pressure [16].

#### 2.2.3. Statistical Analysis

The statistical analysis was performed using R, a free software environment for statistical computing and graphics (the R-fundation, GNU Free Software Foundation, Boston, MA, USA) [17]. The parametric distribution of the variables was analyzed using the Kolmogorov–Smirnov test and residual analysis. Data are presented as mean ± standard deviation (SD), or median (1^st^ and 3^rd^ quartiles), where appropriate. The difference between groups was evaluated with the *T*-test or a three-way ANOVA for normally distributed variables. The Mann–Whitney-Wilcoxon test was used for nonnormally distributed variables, and the *χ*^2^ test or Fisher's exact test was used when appropriate. Correlations among different variables were performed using Person or Spearman's test where appropriate. An alpha-error <0.05 was considered significant in all analysis.

## 3. Results

A total of 41 patients (female 35, 85.3%; mean age: 56.9 ± 13.6 years), 21 with diffuse SSc and 20 with limited SSc were recruited.  Table 1 summarizes anthropometric, clinical, and anamnestic data of the whole population and of the two subgroups of patients with limited and diffuse SSc.

### 3.1. Diffuse vs. Limited SSc

Comparing patients with the two different subtypes of SSc, it clearly showed a similar background: past medical history, familiarity, cardiovascular risk factors, gender distribution, and disease duration were all similar. However, patients with limited SSc were averagely 10 years older than patients with diffuse SSc (51.7 ± 12.7 vs. 62.4 ± 12.5, *p*=0.009). Clinical features were as expected different, with patients with diffuse SSc showing higher prevalence of pulmonary fibrosis, a trend to poorer—although nonstatistically significant—performance on the 6-minute walking test, worse pulmonary function as evaluated by FVC, and an higher MRSS.

Echocardiographic parameters (Table 2) describing potential subclinical cardiac organ damage did not show significant difference between the two subgroups of patients. Left ventricular hypertrophy was found only in 4 of the 41 patients—all female—3 of whom were affected by diffuse SSc (*p* for difference = ns). Tissue Doppler parameters evaluating diastolic function were within normal limit for the majority of the population and showed no significant difference between limited and diffuse SSc. The few patients (*n*=5) that showed abnormal Tissue Doppler parameters were all significantly older than patients showing no abnormalities. Indexed left atrial volume was increased in only 5 patients, again significantly older than the ones showing normal atrial volume, and again with no significant difference between the two subgroups. None of the patients had increased pulmonary pressure values on TTE evaluation.

Patients with limited and systemic SSc also showed similar arterial stiffness, both in terms of cfPWV (6.5 [6–6.8] vs. 7.0 [6.2–8.5] m/sec, *p*=0.24) and Ai (145.6 ± 14.2 vs. 149 ± 20.6, *p*=0.52). A total of 4 patients showed increased (>10 m/sec) cfPWV, with no significant difference in distribution among limited or diffuse form of the disease. These patients were averagely older (73.5 vs. 55.5 years *p*=0.01) but had similar disease duration than the patients with normal cfPWV (*p*=0.27). Dividing patients on the basis of the presence or absence of classical cardiovascular risk factors did not lead to significant difference in the recorded cfPWV, nor cfPWV showed significant difference when the subanalysis was made on the basis of presence of clinical manifestation such as acral ulcers, esophageal hypotonia, pulmonary fibrosis, or positive finding on capillaroscopy (data not shown).

### 3.2. Regression Analysis

In the regression analysis, cfPWV showed a good correlation with age (*r*=0.60, *p* < 0.001) and a fair correlation with disease duration (*r* = 0.36, *p*=0.02). As expected, cfPWV was significantly correlated with both peripheral (SPB: *r*=0.51, *p* < 0.001; DBP: *r*=0.36, *p*=0.02; MBP: *r*=0.45, *p*=0.003) and central blood pressure values (cSPB: *r* 0.49, *p*=0.001; cDBP: *r*=0.37, *p*=0.03; cMBP: *r* = 0.44, *p*=0.003), while Ai did not show significant correlations with any of those variables.

In a multiple regression analysis (Table 3), only age and disease duration maintained a significant correlation with cfPWV after correction for SSc subtype and central and peripheral hemodynamic parameters.

By introducing heart rate and height as independent variables in the multivariate regression analysis, we have found that only age maintained a significant correlation with cfPWV (*p*=0.001), while only height was significantly and inversely correlated with Ai (*p*=0.001) (Table 4).

### 3.3. SSc vs. Controls

Compared to healthy subjects with well-matched hemodynamic profiles (supplemental table S1), SSc patients—irrespective of the clinical subset—did not show a significant increase in arterial stiffness: cfPWV resulted similar in all three groups (6.5 [6–6.8] vs. 7.0 [6.2–8.5] vs. 6.7 [6.2–8] in diffuse SSc, limited SSc, and controls, respectively, *p*=0.458), and no difference in cardiac organ damage, as evaluated with echocardiography, was detected. In a multiple regression analysis mirroring the one performed in SSc patients only, only age maintained significant correlation with cfPWV (*p*=0.008) after correction for disease status (patient vs. controls) and central and peripheral hemodynamic parameters.

## 4. Discussion

In the present study, we evaluated subclinical cardiovascular organ damage in SSc, with particular focus on comparing the two clinical subforms of limited and diffuse SSc, using two standardized evidence-based technologies, TTE and cfPWV.

Echocardiographic evaluation did not show significant prevalence of subclinical organ damage in SSc patients nor significant difference between the subgroups affected by limited or diffuse SSc. Arterial stiffness evaluated with cfPWV showed good correlation with disease duration and patient age. Moreover, cfPWV resulted similar in patients with limited and diffuse SSc, with the latter group of individuals being however, on average, 10 years younger. Disease duration was equivalent in the two subgroups, suggesting that in the diffuse form of the disease, macrovascular damage as assessed by cfPWV and arterial stiffening may be accelerated and more pronounced. However, this hypothesis did not hold true when SSc patients were compared to a well-matched group of healthy individuals that showed similar arterial stiffness parameters.

Cardiovascular diseases are highly prevalent in patients with rheumatic pathologies, such as lupus erythematous and rheumatoid arthritis, and are among the leading causes of mortality in this clinical setting [18–20]. Mechanisms for such a correlation have been identified in accelerated atherosclerosis induced by the chronic inflammation environment and autoimmune milieu [3], closely interplaying with more traditionally recognized cardiovascular risk factors such as hypertension, dyslipidemia, and aging.

In the specific subset of SSc, available data are scanter. However, evidence is increasing in indicating SSc patients at higher cardiovascular risk compared to healthy controls [21]: the European registry indicated indeed that as much as 26% of mortality in SSc patients is related to cardiovascular diseases [22].

The underlying exact pathology has still to be clarified, as well as optimal approaches to screening strategies that may grant early identification of patients at increased risk. In this context, evaluation of subclinical cardiovascular organ damage may represent a pivotal step in patient management. Dedicated techniques, developed in other clinical scenarios, may prove to be a valuable tool. Transthoracic echocardiography able to identify increased left ventricular mass, indexes of diastolic dysfunction, increased left atrial volume [23], and cfPWV measurement [12, 23], evaluating arterial stiffness, are among those.

cfPWV in particular has been recognized as playing a specific role in cardiovascular risk stratification independently of other well-known risk factors [5, 6] in hypertensive patients. Previous studies using this technique have found increased cfPWV values in SSc patients [7, 8] compared to controls, suggesting that arterial stiffness may be part of the disease's multifaced vascular damage. This finding has however not been univocal [9, 10].

In previous work, even when an averagely higher cfPWV value was detected in an SSc patient, the actual number of individuals with pathologically increased arterial stiffness was very low. Moreover, in none of the previous studies was detected a “pathological” cfPWV in SSc patients. Using a cutoff of 9 m/sec, Colaci et al. [8] reported increased cfPWV in older individuals and in patients with prolonged disease duration, hinting a specific role of SSc in inducing arterial stiffening. The used cutoff was however an arbitrary one: threshold values for defining increased cfPWV have been previously published in international guidelines and progressively lowered, from 12 [23] to 10 m/sec [24], but no data have been published on the clinical or prognostic significance of “relatively increased” cfPWV. Even if it may be legitimate to argue that such a threshold has been defined for hypertensive patients and may thus not be automatically applicable to different clinical settings, further investigations are needed in the field before such a conclusion can be drawn. In the same study, a trend for higher cfPWV in limited SSc was also found. However, data on patients' age were not given, so that the aging factor could not be correctly weighted. Age- and SSc subtype-specific evaluation was performed by Timár and colleagues [25] who reported a greater association with increased cfPWV for limited SSc. This finding was not confirmed in the present analysis, in which cfPWV was similar in the two subforms of the disease. However, in the cited study, patients with limited SSc were significantly older—15 years on average—and this may account for the higher cfPWV shown. Indeed, cfPWV is widely recognized to increase with age [26], also in the specific subset of SSc [9, 10].

In the present study, the finding of a comparable cfPWV in the two subpopulations despite an average age difference of 10 years could have been interpreted as an accelerated stiffening of the arteries occurring in diffuse SSc. However, the lack of significant differences in hemodynamic and echocardiographic data, once the SSc population was compared to healthy individuals, weakened such hypothesis. Our data, together with contradicting results from critically reviewed previous studies, point out that traditional tools for the assessment of cardiovascular organ damage—TTE and cfPWV evaluation—might not be the ideal choices for SSc patient risk stratification. Other more specific tools could be better suited to highlight an involvement that might not be expressed neither as arteriosclerosis, in terms of stiffening of the great vessel assessed by cfPWV [10], or classic atherosclerosis, in terms of increased intima-media thickness [26]. For example, alteration of flow-mediated vasodilatation has been proved to occur in SSc patients [27, 28] reflecting endothelial dysfunction, a potential key player in both microvascular and macrovascular involvement.

### 4.1. Limitations

This study shares with other published works on the topic the relatively—as SSc is a rare disease—small sample size and the monocentric design. We acknowledge that the lack of statistical difference between the study groups (limited SSc vs. diffuse SSc vs. control) does not automatically imply similarity, especially within a small population like the one we present here. Indeed, it might be related to the small sample size, and the study itself might be underpowered to thoroughly assess the lack of any significant differences between SSc patients and controls in terms of cfPWV and cardiovascular organ damage. However, the correlation analysis did not show a significant impact of SSc nor its subtype on cfPWV values, highlighting at the same time the importance of age in the determination of arterial stiffness.

Second, in the analyzed population, cfPWV values are mostly within normal limits, with very few patients showing pathologically increased cfPWV (i.e., >10 m/sec) [24]. However, if this small number may be considered to hamper our results, it is nevertheless a real picture of the SSc population, in which increased cfPWV is not indeed very prevalent, supporting our conclusion.

Third, we underline the limitations related to the techniques used in this study to calculate central hemodynamic parameters, since the method to calibrate central pressures may introduce some errors, even if previously validated.

## 5. Conclusions

Echocardiography is one of the tools used for annual screening in systemic sclerosis patients, in order to detect patients at high risk for pulmonary arterial hypertension (PAH) at early stage. For the diagnosis of PAH, the gold standard remains right heart catheterization. The main aim of our paper was to analyze subclinical cardiovascular organ damage in SSc patients and to study in depth the potential role of TTE and cfPWV in cardiovascular risk stratification.

Compared to control, patients affected by SSc did not present significantly increased subclinical cardiac damage nor arterial stiffness as evaluated, respectively, with standard echocardiogram and cfPWV, irrespective of their specific clinical subtypes. The two forms of SSc—limited and diffuse—showed hemodynamic and cardiac features that were similar to those of a well-matched healthy population.

Considering the increasingly growing importance of cost-effectiveness in patient management, our data suggest that cfPWV analysis and echocardiogram may be used in the subsets of SSc patients with additional classical cardiovascular risk factor, better than routinely applied for risk stratification of the general SSc population. Efforts in identifying SSc patients at increased cardiovascular risk should probably be redirected toward use of different technologies.

## Figures and Tables

**Table 1:** Clinical characteristics of the SSc study population.

	All	Diffuse	Limited	*p*
*N*	41	21 (51.2)	20 (48.8)	
Age	56.9 ± 13.6	51.7 ± 12.7	62.4 ± 12.5	**0.009** ^**#**^
Female (%)	35 (85.3)	17 (81)	18 (90)	0.66^$^
Smokers	11 (26.8)	4	7	0.35^$^
Weight (kg)	62.3 ± 10.6	64.5 ± 11.4	59.9 ± 9.4	0.17^**#**^
BMI (kg/m^2^)	24.4 ± 3.7	24.9 ± 3.9	23.8 ± 3.5	0.35^**#**^
SBP (mmHg)	118 ± 17.3	115.9 ± 16.2	121.2 ± 18.3	0.33^**#**^
DBP(mmHg)	69.7 ± 8.6	70.7 ± 7.7	68.6 ± 9.5	0.44^**#**^
HR (bpm)	75 ± 9.7	77.2 ± 9.8	72.9 ± 9.4	0.16^**#**^
*Past medical history*				
Alcohol	8 (19.5)	4 (19)	4 (20)	1^$^
HTN	11 (26.7)	6 (28.6)	5 (25)	1^$^
Hx of HTN	26 (63.4)	12 (57.1)	14 (70)	0.5^$^
Dyslipidemia	8 (19.5)	4(19)	4 (20)	1^$^
Hx of dyslipidemia	14 (34.1)	7 (33.3)	7 (35)	1^$^
DM	1 (0.02)	1 (0.05)	0	
Hx of DM	12 (29.3)	7 (30)	5 (25)	0.73^$^
*SSc-specific parameters*				
Disease duration (months)	81 [46; 130]	60 [34–94]	88 [67; 130]	0.13^$^
***Autoantibodies***				
ANA	39 (95.1)	19 (90.5)	20 (100)	0.48^$^
Scl70	14 (34.1)	14 (66.7)	0	**<0.001** ^$^
Anticentromere	20 (48.8)	0	20 (100)	**<0.001** ^$^
***Clinical features***				
Acral ulcers	15 (36.6)	11 (52.4)	4 (20)	**0.051** ^**$**^
Pulmonary fibrosis	22 (53.6)	15	7	**0.006** ^$^
Renal crisis	1 (0.02)	1 (0.05)	0	
Hypotonia	31 (83.8)	17 (81)	14 (70)	0.67^$^
Positive capillaroscopy	22 (54)	15 (71.4)	7 (35)	0.054^$^

**Table 2:** Echocardiographic and hemodynamic parameters of SSc patients.

	All	Diffuse	Limited	*p*
LVmass (gr)	120 [105–152]	141 [105–184]	122 [102–138]	0.48
LVMi(gr/m2)	79.3 ± 24.8	82.8 ± 27.7	75.7 ± 21.5	0.35
EF (%)	64 ± 4	65 ± 4	64 ± 4	0.08
LVH (#)	4 (9.7%)	3 (19%)	1 (5%)	0.33
LAVi (cc/m2)	23.7 ± 8.1	21.2 ± 7.3	26.5 ± 8.4	0.052
Dilated LA, # (%)	5	1 (5%)	4 (20%)	0.16
E/e' avg	8.4 ± 2.7	7.9 ± 2.1	9.0 ± 3.1	0.22
TR (m/sec)	2.4 ± 0.35	2.2 ± 0.5	2.4 ± 0.2	0.21
pSBP (mmHg)	115 ± 18.7	113 ± 13	117 ± 23	0.5
pDBP (mmHg)	69.3 ± 8.4	69.7 ± 7.6	68.8 ± 9.4	0.73
pMBP (mmHg)	87.4 ± 10.2	86.1 ± 8.2	88.9 ± 11.9	0.39
pAI	88.4 [82.5–99.5]	90 [80.3–95]	93 [82–99]	0.24
cSBP (mmHg)	107.4 ± 17	105.3 ± 11.8	109.7 ± 21.3	0.42
cDBP (mmHg)	70.5 ± 8.5	71.1 ± 7.5	70 ± 9.6	0.66
cMBP (mmHg)	87.5 ± 10	86.1 ± 8.2	88.4 ± 11.7	0.5
cAI	147.3 ± 17.5	145.6 ± 14.2	149 ± 20.6	0.52
CFPWV (m/sec)	6.6[6.1–7.8]	6.5 [6–6.8]	7.0[6.2–8.5]	0.24
CFPWV >10, *n* (%)	4 (9.7%)	2 (9.5%)	2 (10%)	1

**Table 3:** Multivariate regression analysis of cfPWV in SSc patients.

Variables	T value	*p*
Age	2.563	0.0154
BMI	1.237	0.2253
Disease duration	2.072	0.0467
SBP	1.056	0.2991
DBP	−1.277	0.2112
cSBP	0.388	0.7005
cDBP	2.005	0.0537
Clinical subtype	−0.637	0.5287
F 6.31 (<0.001), adjusted *R*^2^ = 0.56		

BMI: body mass index; SBP: systolic blood pressure; DBP: diastolic blood pressure; MBP: mean blood pressure; cSBP: central systolic blood pressure; cDBP: central diastolic blood pressure; cfPWV: carotid-femoral pulse wave velocity.

**Table 4:** Multivariate regression analysis of Ai in diffuse SSc vs. limited SSc vs. controls.

Variables	T value	*p*
Age	−0.263	0.794
Height	−**3.617**	**0.001**
Disease duration	−0.073	0.942
SBP	−1.475	0.146
DBP	1.109	0.272
cSBP	1.773	0.082
cDBP	−1.653	0.104
HR	−1.708	0.093
Clinical subtype	−0.486	0.629

SBP: systolic blood pressure; DBP: diastolic blood pressure; MBP: mean blood pressure; cSBP: central systolic blood pressure; cDBP: central diastolic blood pressure; HR: heart rate; Ai: augmentation index.

## Data Availability

All the data underlying the findings of the study are incorporated in the text and tables.

## Abbreviations

AIx: Augmentation index
DBP: Diastolic blood pressure
cfPWV: Pulse wave velocity
SBP: Systolic blood pressure
SSc: Systemic sclerosis.

## Supplementary Materials

Supplementary Materials S1: clinical and hemodynamic features of SSc patients, divided in the two subtypes of diffuse and limited, and controls.
