# Supplementary material for: Cardiovascular Organ Damage in Clinical Subtypes of Systemic Sclerosis: Arterial Stiffness and Echocardiography Might Not Be the Ideal Tools for Patient Risk Stratification
Source: Cardiol Res Pract. 2021 Apr 23;2021:7915890. doi: 10.1155/2021/7915890 (PMC8087482; doi:10.1155/2021/7915890)
Supplement: Supplementary Materials — S1: clinical and hemodynamic features of SSc patients, divided in the two subtypes of diffuse and limited, and controls. [file 7915890.f1.doc]

**S1 Clinical and hemodynamic features of SSc patients, divided in the two subtypes of diffuse and limited, and controls**

|  | Diffuse SSc  N=21 | Limited SSc  N=20 | Controls  N=23 | p  (ANOVA) |
| --- | --- | --- | --- | --- |
| Age (years) | 51.7 ±12.7 | 62.4 ±12.5 | 58.9 ±5.8 | 0.0069 |
| BMI (Kg/m^2^) | 24.9 ±3.9 | 23.8 ±3.5 | 25.2 ±3.3 | 0.4196 |
| Height (cm) | 161±8 | 159±7***** | 166±8***** | **0.006** |
| SBP (mmHg) | 115.9 ±16.2 | 121.2±18. 3 | 120.1 ±20.0 | 0.5361 |
| DBP (mmHg) | 70.7± 7.7 | 68.6 ±9.5 | 68.1 ±9.0 | 0.5967 |
| HR (bpm) | 77±10***** | 73±9 | 68±11***** | **0.01** |
| LVMi (gr/m^2^) | 86.1 ±29.8 | 79.4 ± 21.5 | 76.2 ± 15.7 | 0.3537 |
| cSBP (mmHg) | 105.3 ±11.8 | 109.7±21.3 | 109.7 ± 10.7 | 0.4576 |
| cDBP (mmHg) | 71.1 ±7.5 | 70± 9.6 | 69.7 ± 8.0 | 0.8462 |
| cMBP (mmHg) | 86.1 ±8.2 | 88.4 ±11.7 | 85.6 ± 7.7 | 0.588 |
| cfPWV (m/sec) | 6.5 [6;6.8] | 7.0 [6.2;8.5] | 6.7 [6.2 ;8] | 0.4528 |
| Aix (%) | 31±7 | 34±7 | 31±11 | 0.41 |

BMI: body mass index; SBP: systolic blood pressure; DBP: diastolic blood pressure; MBP: mean blood pressure; HR: heart rate; LVMi: left ventricular mass index ; cSBP: central systolic blood pressure; cDBP: central diastolic blood pressure ; cMBP: central mean blood pressure ; cfPWV: carotido femoral pulse wave velocity; AIx: augmentation index
